# Osteoclastoma

**Published:** 2008-02

**Authors:** K. Manjunath Rai, S. Venkateswarlu

**Affiliations:** K Manjunath Rai, M.B, FRCR (E), DMR (Lond). Director, Barnard Institute of Radiology, Chennai. From published: and S Venkateswarlu, B.A, MBBS, L.O, DMR Radiologist, Barnard Institute of Radiology, Chennai; M.B, FRCR (E), DMR (Lond). Director, Barnard Institute of Radiology, Madras and B.A, MBBS, L.O, DMR Radiologist, Barnard Institute of Radiology, Madras

Osteoclastoma forms about 20% of all bone tumours. As per the figures of our institute it comes to about 25%. Its incidence before the fusion of epiphysis is not common. It is said to arise in the epiphysis. This view does not find general acceptance; according to this dissenting school, it arises in the metaphysis. In all cases seen by them, the epiphysis is fused and the end of the shaft could not any longer be called the epiphysis. Giant cell tumours occurring before the fusion of the epiphysis were all confined to the metaphysis.

The most common bones involved are the long bones; less common are the jaws, vertebrae, scapula pelvis and small bones of hands and feet. Osteoclastoma is not commonly found in its early stage. It would have existed for sometime before the patient is referred to for roentgenologic examination.

**Figure 1:**
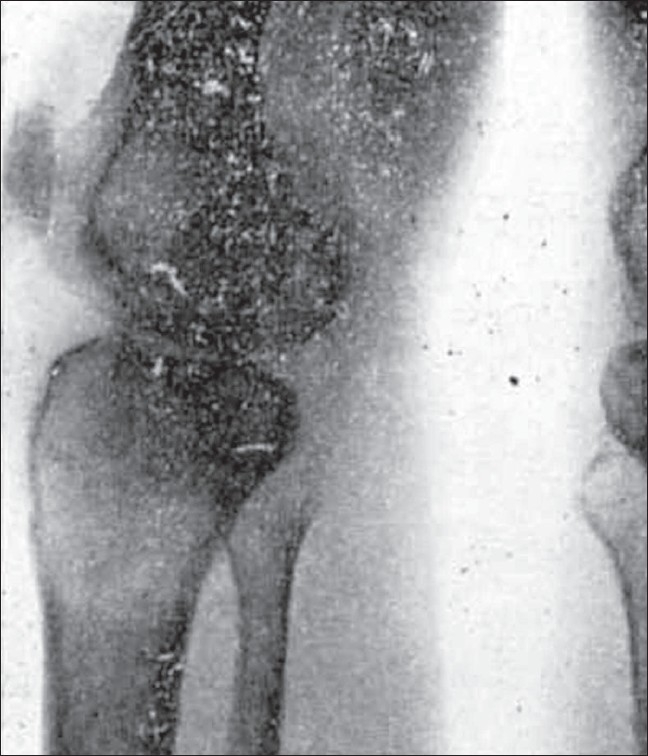
Pictures of an early case of osteoclastoma showing (1) the curved line of graded density and (2) trabeculae. Note there is no expansion yet

Roentgenologic features - The tumour acts essentially as a solvent of bone. It does not infiltrate the bone as a sarcoma does. The line of demarcation between the tumour and the normal bone is clearly defined by a regular convex line, the convexity being towards the normal bone. A characteristic features of this line is its graded density, it is not one dense line [Figure 1]. The density decreases from the normal bone to the area of the tumour. The graded effect is due to the regular process of the dissolution of bone.

**Figure 2:**
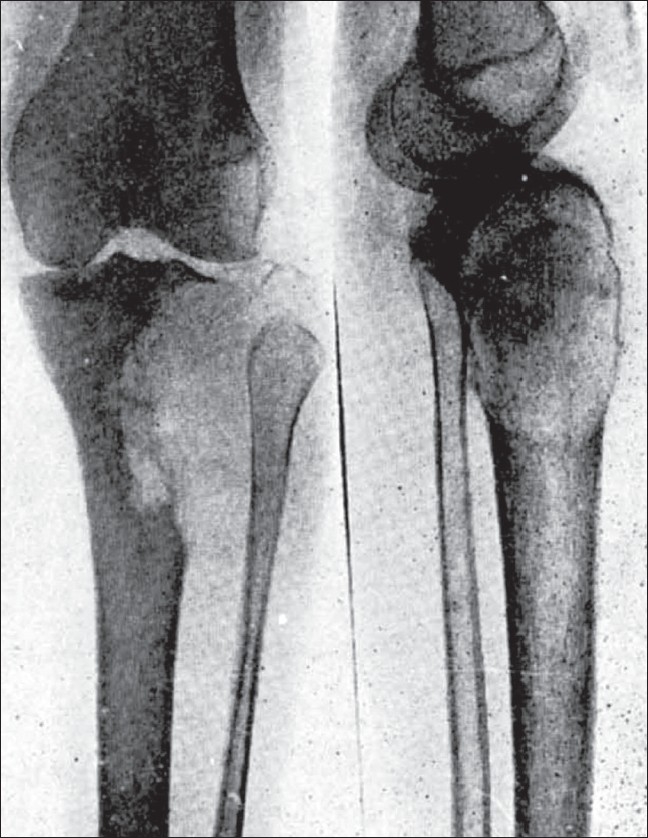
Picture shows (1) Eccentric position. (2) Curved line of graded density. (3) Expansion and extension into the adjacent tissues

**Figure 3:**
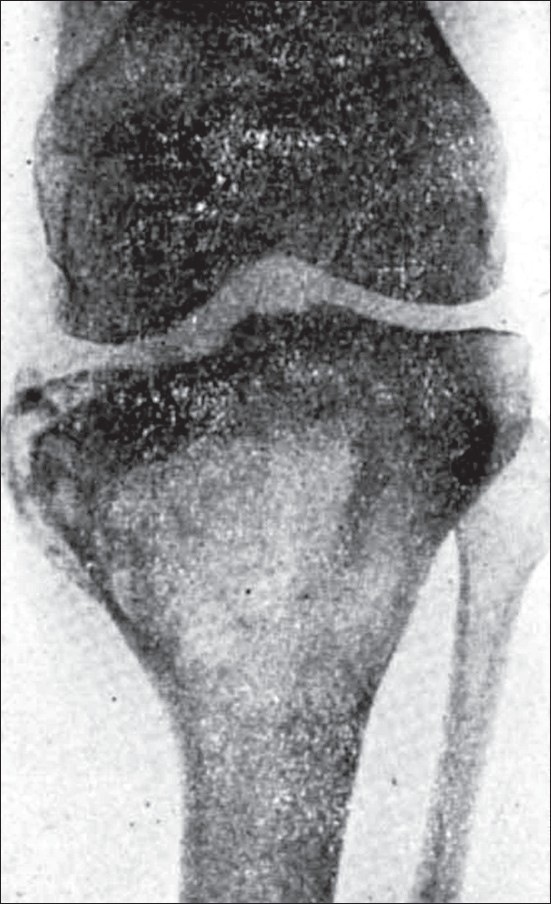
Picture shows the subarticular bone to be intact while the subperiosteal is dissolved

The tumour is usually eccentrically situated; it occupies on side of the bone. It may however start in the centre. In the process of extension the cancellous tissue is gradually dissolved. Later the cortical bone is involved and when it is dissolved and becomes thin begins to bulge due to the expansive nature of the tumour. In the early stages the tumour does not show this expansion. Finally the bony wall is completely absorbed and in some places may break through into the surrounding tissues [Figure 2]. In this respect the subperiosteal bone yields much earlier than the subarticular bone; for a long time the latter shows a protective sclerosis [Figure 3]. The tumour does not show any periosteal reaction. According to some when the cortex becomes a thin shell, it may sometimes be possible to delineate a thin periosteal accretion of new bone.

**Figure 4:**
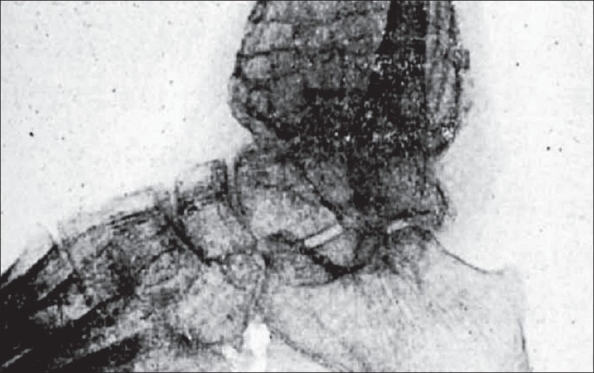
Picture shows well-defined trabeculae

**Figure 5:**
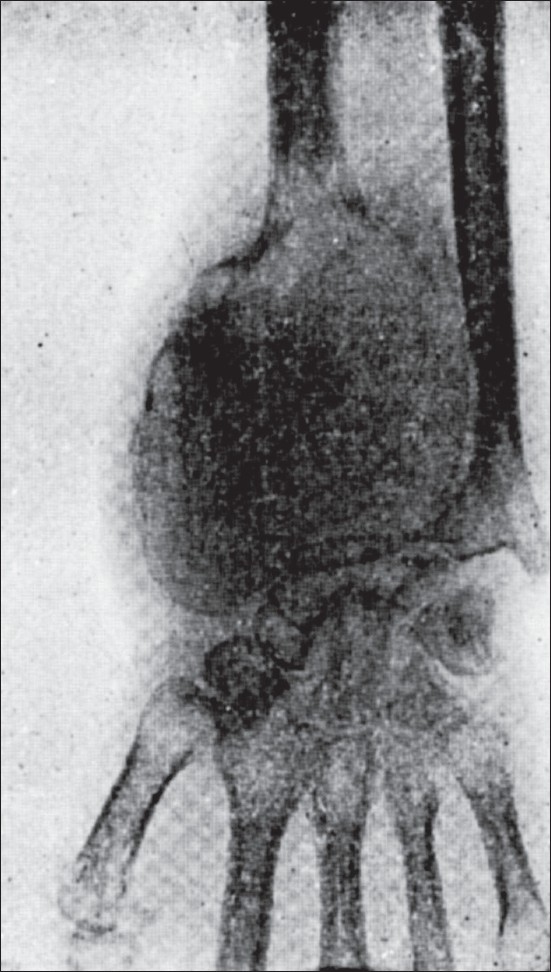
Picture of an actively growing osteoclastoma showing expansion and few trabeculae

**Figure 6:**
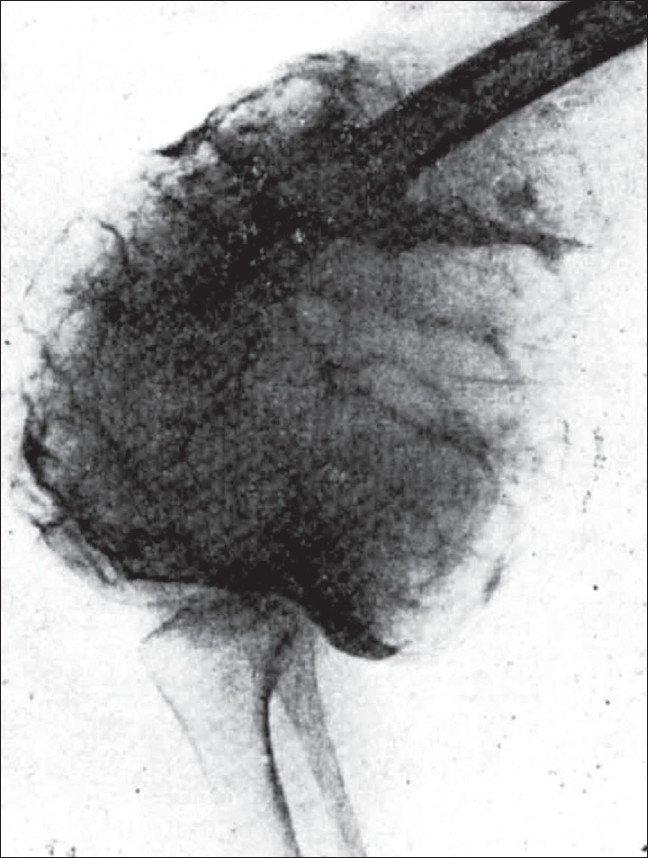
Picture shows gross expansion and “soap-bubble” appearance. The subarticular bone is still intact

In the dissolved area of the tumour a meshwork of bony strands is seen [Figure 4]. These strands are the supporting framework of the delicate cancellous tissue. These also are gradually involved in the process of dissolution. They may be completely absorbed leaving an expanded bony shell [Figure 5]. Often this absorption is uneven and we then have the characteristic soap bubble appearance [Figure 6]. In an actively growing tumour this appearance is not well seen; in these it is evident after irradiation therapy when reossification takes place.

The tumour may extend into the neighbouring bones through fascial planes or ligaments or tendons. It may in some cases cause pressure absorption of adjacent bones. Where bony projections are present near the tumour it grows into them.

To sum up, the radiological features are:

1. Its essential nature of regular dissolution and expansion as compared to irregular destruction and infiltration in osteosarcoma [Figure 7].

**Figure 7:**
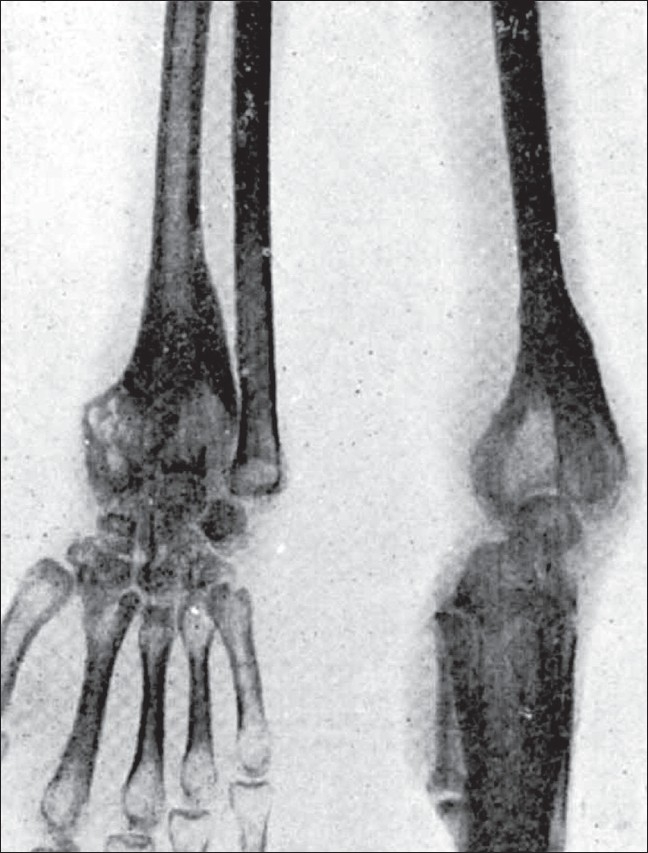
Radiograph of an osteosarcoma. There is expansion; but the abundant periosteal reaction and invasion of shaft with the loss of curved line of demarcation distinguish it from osteoclastoma
2. Eccentric situation in the bone.
3. Curved line of graded density definitely, demarcating the tumour from the normal bone.
4. Absence of periosteal reaction.
5. Extension into adjacent tissue and bones.
6. The relative resistance of the sub-articular bone.

The following characterize the tumour becoming malignant.

1. Invasion into the shaft along the medulla, the curved line of demarcation being no longer well defined.
2. Proliferative reaction of the periosteum.

It is often asked if all these radiological features are infallible; can the surgeon solely depend on these in all cases or should he resort to a biopsy? Our experience does not allow us to answer this question with a simple ‘yes’ or ‘no’. In cases with definite roentgenological findings biopsy is not necessary; where roentgenological findings are not definite and clinically malignancy is suspected a biopsy is advised.

Differential diagnosis - The following conditions have to be differentiated from osteoclastoma:

1. Plasmacytoma
2. Adamantinoma
3. Osteitis fibrosa of focal and generalized types
4. Chondromata
5. Bone cysts
6. Osteolytic sarcoma
7. Metastatic carcinoma
8. Cystic type of tuberculosis

Differentiation in certain cases is not possible only from radiological findings; clinical features have also to be taken into consideration. Sometimes it is necessary to repeat the roentgenologic examination at frequent intervals, so that the progress and nature of the lesion may be known.

*Plasmacytoma:* The solitary type is to be differentiated. The trabeculae are few, well-defined and coarse; urine may show Bence-jones protein and sternal puncture, plasma cells.

*Adamantinoma:* The line of demarcation from the normal bone is sharp, the trabeculae are coarse and sometimes the tumour gives a honeycombed appearance. It is not radiosensitive.

*Focal osteitis fibrosa:* Though usually solitary other bones may show similar lesions, trabeculae are few and course, outline is sharp and progress is slow.

*Generalized osteitis fibrosa:* There is generalized osteoporosis; blood chemistry shows changes and deposition of calcium in urinary tract may occur.

*Chondramo:* It may be anywhere in the shaft, has a well-defined and clear cut boundary and on radiographic examination of the skeleton similar lesions may be found in other bones.

*Bone cyst:* It has a clear cut sclerosed margin and may be situated anywhere in the shaft.

*Osteolytics sarcoma:* There is no definite line of demarcation from normal bone; periosteal reaction and invasion of the tumour into the shaft occur.

*Metastatics carcinoma:* The secondary deposits from thyroid have a soap-bubble appearance and this has to be borne in mind. Age and site as also the history or presence of primary lesion help in differentiation. Urine may show Bence-Jones protein. These lesions have invasive and destructive properties.

*Cystic type of tuberculosis:* Repeated examination at short intervals shows the cysts to coalesce; the articular surface later breaks down and ultimately the joint may be destroyed.

We would like to draw attention to the following features of diagnostic importance:

1. Arteriographic findings
2. Characteristics pain of osteoclastoma.

**Figure 8:**
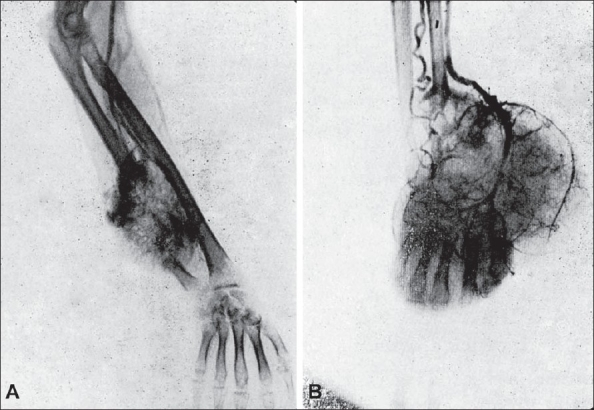
Arteriograms of osteosarcoma and osteoclastoma. (A) Osteosarcoma - shows great increase in the arterioles and venules in the area of the tumours. (B) Osteoclastoma - though there is increased vascularity, it is definitely not the picture seen in osteosarcoma - no increase in arterioles and venules

Arteriography shows a very great increase in the number of arterioles and venules both inside and outside the bone in malignant disease [Figure 8A]. This does not happen in any other bone disease. In osteoclastoma though there is increased vascularity, there is never the picture of increased arterioles and venules as in osteosarcoma [Figure 8B]. The pain in osteoclastoma is of a dull-boring type, in contradistinction to the very severe pain seen early in osteosarcoma. There is one characteristic feature about this pain; under irradiation therapy pain of osteoclastoma does not disappear soon, whereas in osteosarcoma relief of pain occurs very early.

**Treatment:** It is either surgical curettage or radiation. Surgical procedure had been the sheet-anchor for a long time till it has been found that this tumour is radiosensitive. For a time both methods were combined with the hope that recurrences after curettage, which were not infrequent could be prevented. In fact we followed this method ourselves; in the earlier days the results looked satisfactory, but later with the added experience of treating larger number of cases, we have learnt to our cost that this combined treatment of surgical curettage followed by irradiation is dangerous. The effect of this combined therapy is (1) there is delay in repair as irradiation causes damage to newly formed capillaries; (2) the tumour may become malignant. The increased irritation due to the double trauma of surgery and irradiation is supposed to be too much and is considered to be a factor in transforming a benign tumour into a malignant one, which accident is not infrequent in this combined method.

Though everybody is agreed that either surgery or irradiation is the line of treatment, there does not seem to be any definite agreement among surgeons and radiologists as to which is to be preferred. The surgeon claims that osteoclastoma requires his instrument and deft fingers; the radiotherapist is equally jealous and claims its treatment to come under the prerogative of his speciality. The choice between these two lines of treatment should depend on certain factors. Some cases are treated only by radiation, some only by surgery, while certain others are suitable for either method. The factors which determine the line of treatment are: (1) age, (2) site, and (3) extent. In young individuals where the epiphyseal union has not taken place surgery is the method of choice; irradiation in these cases by its effect on endochondral ossification interferes with the growth of the bone and so is contraindicated. In certain inaccessible sites as vertebra and pelvic bones, surgery is not possible. In extensive tumours with gross involvement of bone, radiation is the method of choice as pathological fracture may occur after curettage. Small tumours where the extent of involvement is moderate are suitable for either surgery or irradiation but we prefer irradiation. In tumours extending into the joints, it is said that surgery is to be preferred but we see no reason why radiation should not be used; we have had occasion to treat such cases with irradiation with good results. In tumours of the digit of hands and feet surgery is preferred in as much as the treatment is finished quicker.

**Figure 9:**
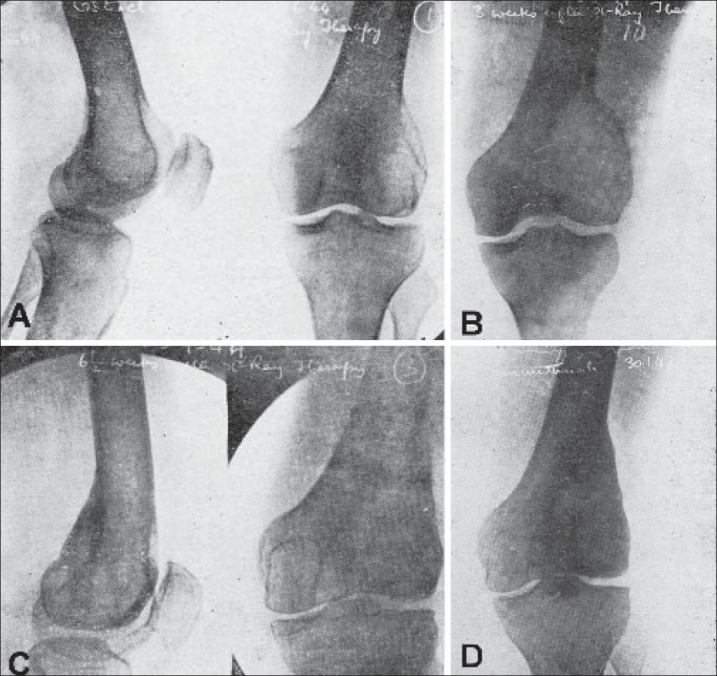
Effects of Irradiation in a case of osteoclastoma. (A) Before Roentgen irradiation. (B) 3 weeks after irradiation - Note increase in size and osteolysis. (C) 6½ weeks after irradiation - consolidation has commenced. (D) 2½ years after complete consolidation. (The patient failed to report between 6½ months and 2½ years and so pictures could not be obtained in this period

**Figure 10:**
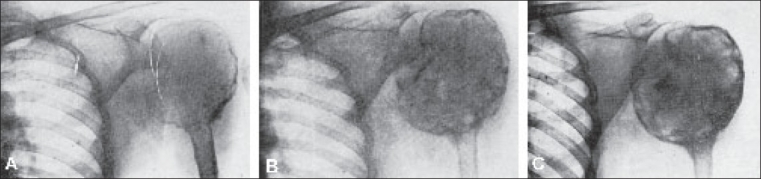
Effects of X-radiation on osteoclastoma. These pictures show progressive recalcification. The trabeculae which were not seen in the pictures before irradiation are well brought out when reactive bone is laid down after irradiation. (A) Before irradiation, (B) 4 months after irradiation, (C) 7½ months after irradiation

**Figure 11:**
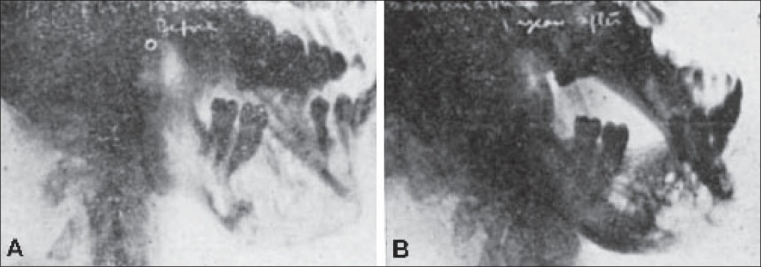
Effects of Irradiation in a case of osteoclastoma of the Mandible. There is complete consolidation of tumour with regularisation of the anatomical contours. (A) Before irradiation, (B) One year after irradiation

**Figure 12:**
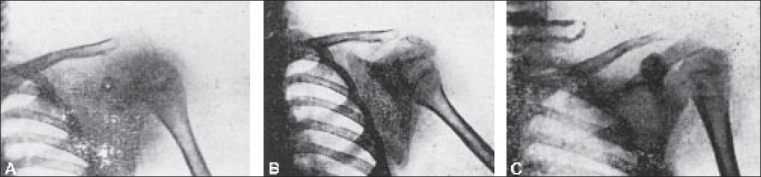
Pictures of osteosarcoma of scapula treated with X-ray therapy. (A) Before X-ray therapy, (B) After 2,000r (C) After 5,000r

**Figure 13:**
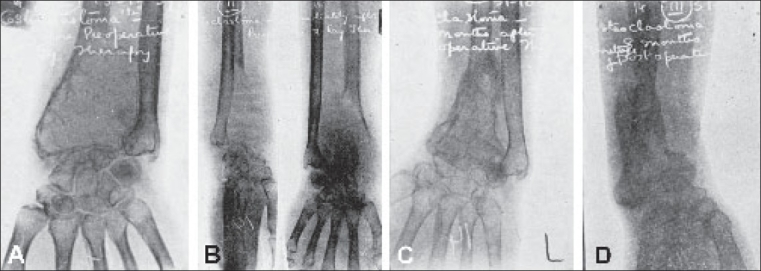
Osteoclastoma treated by the old combined method of irradiation and surgery. (A) Before irradiation (pre-operative), (B) Immediately after irradiation, (C and D) 8 months after curettage and post-operative irradiation. There is good consolidation; the condition has not recurred till today-nearly 7 years since treatment. (This method of treatment has since been given up)

**Radiation therapy:** The type of radiation recommended is roentgen therapy. We use only this method. According to BRAILFORD its offers good results in about 85% of the cases. Complete consolidation is obtained by this method alone. Functional result is better than with surgery. Even in tumours with gross involvement of bone where amputation seems inevitable, radiation therapy has proved of great avail leading to consolidation of the diseased area and saving the loss of limb. The dose required is not very high. We give a tumour dose of 2,000 roentgens delivered in a period of about four weeks. The factors are 200 kV, 0.5 mm of Cu plus 1.0 mm of AI filter, 50 cm F.S.D 150r at a sitting; treatment is on alternate days. There is no necessity for repetition of the treatment. When high doses are used it takes longer time to calcify; nothing else happens. Certain changes which happen under irradiation should be noted [Figures 9–13]. Immediately after the treatment there is an “apparent extension” of the tumour. Skiagrams taken 4-8 weeks after treatment show this; there is more of osteolysis. Later the tumour area commences to consolidate; recalcification takes place and in a period extending from 8 to 10 months the consolidation is completed. During the period when the skiagrams show increased osteolysis, clinically too the tumour appears bigger red and glossy. This often mistaken as activation of the tumour by those not conversant with radiation therapy and had been the cause of many an amputation. The increased osteolysis seen in osteoclastoma immediately after treatment is not feature of osteosarcoma; on the other hand after irradiation the latter tumour shows evidence of progressive shrinkage and recalcification. During the period of osteolysis the limb must be immobilized if it is a weight bearing one.

In treatment it is sometimes a matter for decision as to whether a particular tumour is benign and to be treated as such or is malignant and requires to be drastically dealt with. For the experienced radiologist it should not be a matter of difficulty, nevertheless there are cases highly suggestive of malignancy. In these it is advisable to do a biopsy. This does not in any way hamper the chances of successful treatment. The latest method of treatment of bone sarcoma is irradiation followed by amputation. The five year survival figures of New York Memorial Hospital, as quoted by Prof. CADE are as follows - “with amputation alone 4.5%; with irradiation followed by amputation 46%”; this being the case the best procedure in an osteoclastoma suspected to be undergoing malignancy would be to do a biopsy and knowing its nature follow it up with irradiation if it is benign, or irradiation and amputation if it is malignant. There is no need for a biopsy in an osteoclastoma, the radiological features of which are precise. BRAILSFORD goes to the extent of advising against biopsy in every case. In his view “there is no simple lesion which produces this radiographic appearance which will be improved by biopsy and there is no malignant lesion which will not be the better for radiation, even if amputation appears to be desirable.

